# Botulinum toxin-A for the treatment of overactive bladder: UK contributions

**DOI:** 10.1177/2051415812473096

**Published:** 2013-03

**Authors:** JH Seth, C Dowson, MS Khan, JN Panicker, CJ Fowler, P Dasgupta, A Sahai

**Affiliations:** 1 Department of Urology, Medical Research Council (MRC) Centre for Transplantation and Guy’s and St Thomas’ NHS Trust, King’s College London, King’s Health Partners, Guy’s Hospital, UK; 2 Department of Uro-Neurology, National Hospital for Neurology and Neurosurgery, UCL Hospitals Foundation Trust, UK

**Keywords:** Botulinum toxin A, idiopathic detrusor overactivity, neurogenic detrusor overactivity, urinary incontinence, quality of life

## Abstract

**Background:**

Botulinum toxin-A (BoNT/A) is now established second-line management for refractory overactive bladder (OAB) and recognised in many incontinence guidelines and pathways. For those with neurogenic detrusor overactivity secondary to spinal cord injury or multiple sclerosis, the toxin is currently licensed in certain parts of the world, including the UK. It is an effective treatment in those in whom antimuscarinics and conservative measures have failed who have symptoms of OAB and or detrusor overactivity (DO).

**Methods:**

Treatment can be given in an outpatient setting and can be administered under local anaesthesia. Its efficacy lasts for between six and 12 months.

**Results:**

It has an acceptable safety profile with the biggest risk being urinary tract infection and difficulty emptying the bladder, necessitating clean intermittent self-catheterisation (CISC). Medium-term follow-up suggests repeated injections are also safe and efficacious.

**Conclusions:**

The mechanism of action of the toxin is more complicated than originally thought, and it seems likely that it affects motor and sensory nerves of the bladder. In the last 10 years much of the progress of this treatment from early experimental trials to mainstream clinical use, and a better understanding of how it works in the bladder, are as a result of research conducted in the UK. This review summarises the significant and substantial evidence for BoNT/A to treat refractory OAB from UK centres.

## Introduction

The use of botulinum neurotoxin type A (BoNT/A) has become a recognised second-line treatment modality for symptomatic patients with idiopathic (IDO) and neuropathic detrusor overactivity (NDO). In the United Kingdom (UK) the treatment is administered in those for whom conservative measures and antimuscarinics have failed, as stated in the National Institute for Health and Clinical Excellence (NICE) guidance on urinary incontinence (UI) in women.^1^ Its use is also recommended by the European Association of Urology (EAU)^2^ and was given a grade A recommendation by an expert panel from Europe related to its efficacy.^3^ Furthermore, a large comprehensive systematic review by Mangera et al. confirmed that the toxin was efficacious in various forms of lower urinary tract dysfunction, including detrusor overactivity (DO).^4^ As there are different formulations and different companies manufacturing and marketing BoNT/A, the United States (US) Food and Drug Administration (FDA) have revised the nomenclature for the toxin, especially as dosing between Botox® (Allergan, Ltd, Irvine, USA) and Dysport® (Ipsen, Ltd, Paris, France) are different. Botox® is now known as onabotulinumtoxin A and Dysport® as abobotulinumtoxin A.

Onabotulinumtoxin A has been approved for urological use in the bladder to treat NDO secondary to spinal cord injury (SCI) or multiple sclerosis (MS) in certain parts of the world, including the US and the UK. Soon-to-be-reported-on phase III pivotal multi-centre clinical trials will facilitate its licensing in IDO in the future.

This article highlights the research contribution from the UK, which is significant, on this remarkable toxin and its use in the bladder.

## Mechanism of action

The effect of BoNT/A on inhibiting parasympathetic presynaptic release of acetylcholine (ACh) at the neuromuscular junction is well known. BoNT/A neurotoxin binds to peripheral cholinergic terminals and inhibits ACh release at the neuromuscular junction. Four steps are involved in this process: binding, translocation, cleavage and inhibition of transmitter release, resulting in blockage of synaptic transmission and flaccid paralysis in the target muscle ensues^5^ (Figures 1 and 2).

**Figure 1(a).**
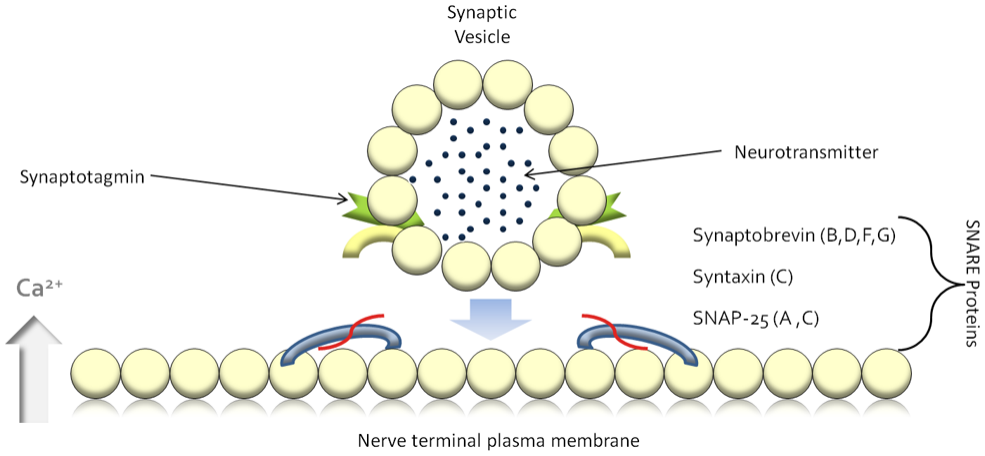
For exocytosis to occur SNARE proteins are required to anchor the acetylcholine-containing vesicles to the neuronal membrane. SNARE: soluble N-ethylmaleimide-sensitive-factor attachment protein receptor; SNAP-25: synaptosomal-associated protein 25.

**Figure 1(b).**
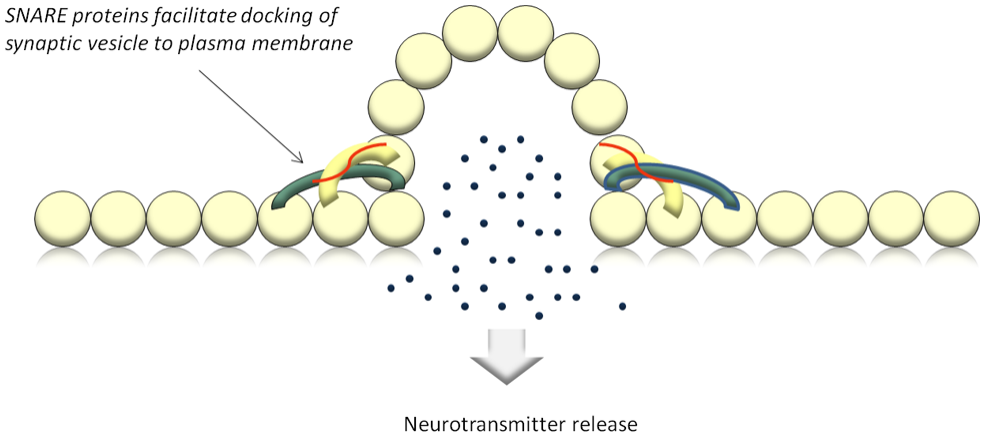
Acetylcholine is released into the neuromuscular cleft where it will bind to muscarinic receptors on the muscle end plate and cause contraction. SNARE: soluble N-ethylmaleimide-sensitive-factor attachment protein receptor.

**Figure 2(a).**
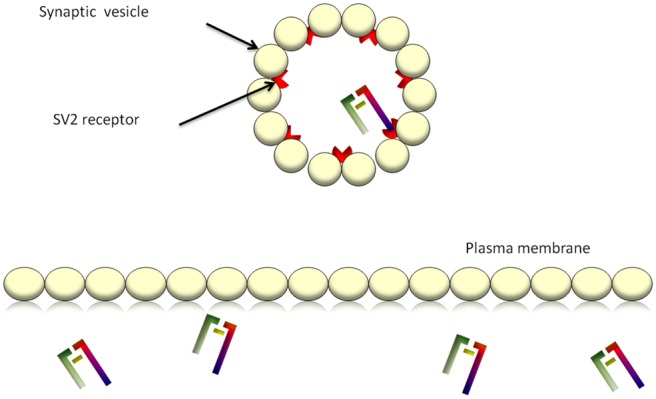
BoNT/A binds to the SV2 receptor and is internalised. The heavy chain of the toxin facilitates entry. BoNT/A: botulinum toxin-A; SV2: synaptic vesicle glycoprotein 2.

**Figure 2(b).**
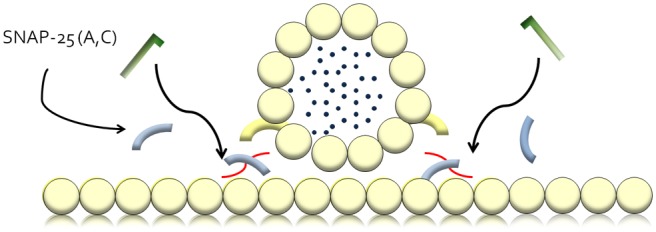
The light chain of the toxin acts as an enzyme and cleaves the various SNARE protein components. In the case of BoNT/A, it cleaves SNAP-25, which prevents acetylcholine release and hence flaccid paralysis ensues in the target muscle.SNARE: soluble N-ethylmaleimide-sensitive-factor attachment protein receptor; BoNT/A: botulinum toxin-A; SNAP-25: synaptosomal-associated protein 25.

A dual mechanism of action for BoNT/A on both the efferent and afferent arms of the micturition reflex has been proposed.^6^ Studies of human bladder biopsies taken at four and 16 weeks following onabotulinumtoxin A injections have shown a reduced expression of vanilloid (TRPV1) and purinergic (P2X_3_) receptors in the sub-urothelium of patients with NDO and IDO.^7^ Both these sensory receptors are up-regulated in IDO and NDO at baseline, and their levels by 16 weeks normalise to that of controls following administration of either 200 or 300 IU of onabotulinumtoxin A.

A potential concern with repeated BoNT/A injections is bladder wall fibrosis and its sequelae. Bladder biopsies were taken before and four and 16 weeks after the first and repeated injection of BoNT/A.^8^ A total of 179 biopsies from 79 patients were analysed for inflammatory changes, fibrosis, hyperplasia and dysplasia. The severity of inflammation remained unchanged, even after repeat injections of onabotulinumtoxin A. Equal levels of fibrosis (2.2%) were seen pre- and post-injection. No evidence of dysplasia or hyperplasia were detected and no significant difference existed between NDO or IDO.

BoNT/A, when injected into the prostate, has been shown to reduce prostate size by apoptotic mechanisms.^9^ As a result BoNT/A has been trialled in humans to treat benign prostatic hyperplasia and is reviewed elsewhere.^10^ To assess whether apoptosis is significant for the mode of action in the bladder, biopsies were taken from 12 patients with NDO secondary to multiple sclerosis and seven healthy controls, before and four weeks after onabotulinumtoxin A injections. Identification of apoptotic cells was performed using terminal deoxynucleotidyl transferase-mediated dUTP nick-end labelling (TUNEL) staining. No difference between the two groups was demonstrated, suggesting that apoptosis is not likely to be a significant factor contributing to the mechanism of action in the bladder.^10^

Recent evidence suggests interstitial cells of Cajal-like cells (ICC) in the bladder suburothelium may act as ‘mechano-receptors’ with implications in the pathophysiology of DO.^11,12^ In the bladder, these cells form a matrix, separated by gap junctions, extensively coupled with connexin 43 (Cx43), and closely associated with the afferent nerves on electron microscopy. A study assessing suburothelial biopsies suggested Cx43 was increased in NDO and IDO when compared with control biopsies; however, this remained unchanged after BoNT/A injections.^13^ No significant differences were seen in ICC marker (vimentin, c-kit) immunoreactivity when related to controls or BoNT/A administration. The authors concluded that the beneficial effect of BoNT/A in suppressing DO is unlikely to be caused by remodelling of the gap junction distribution, at least in the suburothelium.

Datta et al. recently assessed muscarinic receptors in the human urothelium and suburothelium and the effects of onabotulinumtoxin A.^14^ The expression of muscarinic receptors was decreased in patients with DO. Successful DO treatment with onabotulinumtoxin A appeared to normalize M1, M2 and partly M3 receptor levels. Baseline and post-treatment changes in these muscarinic receptor levels were inversely associated with patients’ overactive bladder (OAB) symptoms and increased with an improvement in patient symptoms. Concomitant antimuscarinic use did not seem to affect results. The authors concluded, however, that the functional significance of such results remain as yet to be determined.

## Technique of injection

The original description of BoNT/A injections for the treatment of NDO was through a collagen flexible needle using a rigid cystoscope.^1,15^ Utilising this technique, a magnetic resonance imaging (MRI) study in six patients following BoNT/A injections mixed with contrast showed the majority of the toxin to be in the detrusor muscle (82%) but some of the contrast material was seen to be situated in the perivesical fat tissue outside the detrusor. The technique employed utilised a rigid cystoscope and a 22 G needle and had a needle length of 8 mm which was inserted into the bladder wall and withdrawn halfway prior to injection.^16^ In an open-label study using onabotulinumtoxin A for NDO and IDO patients commenced in 2002,^17^ clinicians began exploring an alternative method of delivering the treatment using a flexible cystoscope and an ultra-fine 4 mm length flexible needle (Olympus, Keymed, UK) performing injections in an outpatient setting under local anaesthesia.^18^ The objective was to ensure that the toxin could be delivered at an optimal depth into the sub-mucosa or detrusor muscle, but not beyond. A fine sheath (27 G) was introduced through the working channel of the cystoscope and the ultra-fine needle was passed through this sheath. This provided needle stability, injection precision and protection to the cystoscope. As the ultra-fine needle is buried into the bladder mucosa, a depth of 4 mm will not be exceeded and the chance of backflow of the toxin after removal of the needle is reduced. Several companies have developed their own injection needles in light of the recent licensing of onabotulinumtoxin A in NDO, all with slightly different characteristics. The exact location of injection and at what depth, injection number and volume of injection have not been standardised, with some arguing this is unlikely to alter efficacy in a significant way. However, a recent study by Manecksha et al. looked at trigone-inclusive (20 injections — five in the trigone and 15 outside the trigone in the bladder) versus trigone-sparing (20 injections throughout the bladder) abobotulinumtoxin A (500 IU total) injections into the bladder to treat refractory OAB.^19^ Utilising the overactive bladder symptom score (OABSS), significant reductions were seen in the overall score and in the urgency subscale in favour of the trigone-inclusive technique.

## NDO

Popat et al. in an open-labelled study compared the effect of onabotulinumtoxin A injections at 200 IU for IDO and 300 IU for NDO.^3,17^ Significant improvements were demonstrated in IDO as well as NDO for OAB symptoms, urodynamics and quality of life (QoL);^20^ however, urgency was significantly better with NDO compared to IDO at four and 16 weeks. When looking at IDO and NDO patients as a whole, Kalsi et al. found that the improvement in QoL correlated well with improvements in OAB symptoms but not with urodynamic parameters.^20^ The speed of the effect was obvious to patients and clinicians. Kalsi et al. have reported on NDO patients treated with 300 IU onabotulinumtoxin A and assessed patients with a seven-day voiding diary immediately after injection.^21^ Significant improvements compared to baseline were demonstrated as early as two days for urgency, frequency and nocturia and by day 3 for urge UI.

Patki et al. utilised abobotulinumtoxin A in their series of 37 SCI patients and concluded that 1000 IU was effective in treating NDO, with improvements in QoL scores and urodynamic parameters. Additionally 50% of patients were able to stop antimuscarinic medication.^22^ However, two cases of transient muscle weakness were observed with this dose. The mean duration of benefit was approximately nine months in this study. The same group also reported on patients’ satisfaction in a group of patients with SCI and NDO also treated with abobotulinumtoxin A.^23^ In this study mean patient satisfaction scores were 6.2/10, and 90% of patients selected the option to have BoNT/A treatment as their long-term treatment option. Only 15% wished to consider a permanent alternative solution such as augmentation cystoplasty.

Large numbers of studies have been conducted examining the effect of BoNT/A in patients with various causes of spinal cord dysfunction especially after spinal injury. Relatively little was known about patients with MS, which can cause debilitating bladder symptoms. A prospective study of 43 patients with MS who suffered from severe urge incontinence demonstrated the efficacy of onabotulinumtoxin A in this setting.^24,25^ Patients were followed up at four and 16 weeks, with significant improvements found in QoL and urodynamic parameters, with a mean duration of effect of 9.7 months. Although this effect was sustained with repeat treatments, it was noticed that 98% of patients had to perform CISC after treatment.

The recent licensing of onabotulinumtoxin A has been on the background of recent company-sponsored multicentre phase III randomised controlled trials, of which one involved several UK centres.^26^ Two hundred and seventy-five patients were randomised to onabotulinumtoxin A at 200, 300 U and placebo. Patients with MS and SCI who had ≥ 14 UI episodes per week were recruited. The primary endpoint of UI episodes at six weeks was significantly reduced with onabotulinumtoxin A at both doses compared with placebo. At this timepoint fully continent rates were 7.6%, 38% and 39.6% for placebo, 200 U and 300 U, respectively. Incontinence QoL scores were also significantly greater at six weeks, suggesting improved QoL in those treated with onabotulinumtoxin A compared with placebo. The median duration of effect of onaboutlinumtoxin A was 42.1 weeks in contrast to placebo, which was 13.1 weeks. Seventy-four patients received a second injection with similar benefits. Approximately 50% were not performing CISC at baseline. The need to instigate CISC was 12%, 30% and 42% in the placebo, 200 U and 300 U groups, respectively. The study concluded 200 U was equivalent to 300 U but with a better safety profile. Although typically open-labelled studies utilised 300 U for treating NDO, on the basis of the phase II and III clinical trial data it is likely the initial recommended dose for this patient population will be 200 U onabotulinumtoxin A.

## IDO

In an open-labelled study, Kalsi et al. looked at when OAB symptoms change following onabotulinumtoxin A injections at 200 IU in IDO patients.^21^ The study showed frequency, urgency and urge UI was significantly reduced by day 4 following treatment. Nocturia took longer to reduce significantly. Urgency remained significantly reduced after day 4 up until week 4 but frequency and urge UI were a little more variable, although by week 4 all OAB symptoms including nocturia were significantly less compared to baseline. In another study assessing IDO and NDO patients, the same institution reported on significant improvements in QoL as assessed by the Urogenital Distress Inventory-6 (UDI-6) and Incontinence Impact Questionnaire-7 (IIQ-7) at four and 16 weeks post-treatment compared to baseline.^20^ This study found a statistically proven correlation between improvement in QoL with improvements in urgency and urge UI for IDO and NDO patients and also frequency in the NDO population alone. Khan et al. have recently published data on patient-reported outcomes of incontinence using two out of the six questions on the UDI-6.^27^ In this open-labelled study in which the data were collected four weeks’ post-injection in 74 patients, complete continence rates in previously incontinent patients were reported as 51%. In all patients, including those for whom complete continence was not achieved, significant improvements in OAB parameters were still observed.

Another study from the UK, the first to report on the use of Dysport® in IDO, utilised 500 U of abobotulinumtoxin A with significant benefit.^28^ In this study all patients had urge UI pre-injection. Sixty-three per cent of patients were dry at one week and 32% at three and six months. Significant reductions in frequency and urgency were seen for up to six months compared to baseline. Pad usage was significantly less after six weeks of treatment. Although trends of improvement were seen in urodynamic parameters, only first desire to void was significantly increased in BoNT/A-treated patients at three months. DO seen in 100% pre-injection had resolved in 40% by three months. High rates of voiding dysfunction were seen, with 35% requiring a suprapubic catheter or CISC at six weeks’ follow-up.

The first double-blind placebo-controlled trial with onabotulinumtoxin A and IDO patients refractory to antimuscarinics was reported in 2007.^29^ Patients were randomised to 200 U (Botox®) (*n*=16) or placebo (*n*=18) administered using a flexible cystoscopic technique under local anaesthetic. The primary endpoint, maximum cystometric capacity, increased significantly, and improvement in symptoms, urodynamic and QoL parameters using the UDI-6 and IIQ-7 was seen in patients in the BoNT/A group compared with placebo. Unblinding took place after 12 weeks, and data from the open-labelled extension study suggested the beneficial effects lasted at least for 24 weeks. Six patients, all in the onabotulinumtoxin A group, had symptomatic >150 ml residual volume at follow-up and were taught CISC. Further studies have confirmed that at this dose the incidence of CISC is approximately 40%.^24,30^ A larger multicentre, randomised, double placebo-controlled trial of 240 female patients with refractory IDO (RELAX study), among eight UK urogynaecology centres, has been published also confirming the efficacy of onabotulinumtoxin A at 200 U.^31^ Outcomes such as voiding frequency per 24 hours, urgency and incontinence episodes were all significantly improved in the treatment arm over placebo. Continence was more common following toxin treatment compared to placebo, 31% vs 12%. Rates of UTI and self-catheterisation were also significantly raised in the treatment groups at 31% and 16%, respectively. Finally a dose-escalation multicentre company-sponsored trial involving UK centres has reported that at week 12 mean change from baseline in urinary urgency incontinence (UUI) episodes was −17.4, −20.7, −18.4, −23.0, −19.6 and −19.4 for the placebo and onabotulinumtoxin A dose groups of 50, 100, 150, 200 and 300 U, respectively. Dry rates were 15.9%, 29.8%, 37.0%, 40.8%, 30.9% and 57.1% in the placebo, and 50, 100, 150, 200 and 300 U dose groups, respectively. Although a clear placebo effect is seen, there were statistically significant differences between active treatment and placebo at various timepoints. Using non-parametric analysis and a rank residual score, a dose-dependent effect was observed with minimal additional benefit for this parameter with doses > 150 U. The lowest dose of 50 U did not appear to be as effective as doses 100–300 U. Those with DO or not had similar benefits. Dose-dependent increases in post-void residual urine volume (PVR) were observed up to 200 U. The maximal effect of increased PVR was at two weeks and thereafter values declined to 36 weeks. Adverse events reported significantly higher in the onabotulinumtoxin A groups compared with placebo were high PVRs and urinary tract infection (UTI). The percentage of patients requiring an indwelling catheter or CISC were 0, 5.4, 10.9, 20.0, 21.2 and 16.4% for placebo, 50, 100, 150, 200 and 300 U, respectively. The phase III data are currently awaited but it is likely that onabotulinumtoxin A doses of 100–150 U will be recommended in the first instance.

As experience with using BoNT/A to treat OAB increased, clinicians became aware of its significant benefits but also of some limitations. Patient selection was crucial and furthermore not every patient had a good or excellent response. Some patients did not respond to treatment and some potential drawbacks such as voiding dysfunction necessitating CISC, which were far more prevalent than originally described, became apparent. In one study assessing poor IDO responders, high maximum detrusor pressures (MDP) during filling cystometry of > 110 cm H_2_0 was predictive of a poor response to treatment with 200 U onabotulinumtoxin A.^32^ In such patients, the authors reported, with higher doses successful outcomes were possible. The same group also assessed the need for CISC following BoNT/A and found simple-to-calculate detrusor contractility variables from urodynamic data such as projected isovolumetric pressure-1 and bladder contractility index may be helpful in predicting CISC and be helpful in counselling patients.^33^ However, these findings need to be confirmed in larger-scale studies. This study as well as another study assessing onabotulinumtoxin A at 300 U in female patients with IDO have shown detrusor contractility does reduce following treatment, confirming its likely effect on efferent blockade. Interestingly, improvements in the QoL offered by onabotulinumtoxin A remain despite the need for CISC. In a prospective study, 65 women with refractory IDO were treated with 200 IU.^34^ No significant differences in the degree of QoL score improvement were seen pre- and post-treatment between those who required CISC and those who did not. The group concluded that there is no impairment to the QoL in those who require CISC after onabotulinumtoxin A once appropriately informed of the risks.

An interesting retrospective study at a large UK teaching hospital followed up patients up to 60 months after their first injection.^35^ From this analysis, it appeared that almost two thirds of patients (61.3% to 63.8%) had discontinued BoNT/A treatment by 36 to 60 months, respectively. The main reasons for this were tolerability issues due to the need for CISC and UTI. Loss of efficacy was of secondary importance in these patients. This study highlights the possible issues of real-life practice outside clinical trials, and in their hands drop-out rates are high with patients pursuing other treatments, such as conservative, neuromodulation and surgical interventions. However, Dowson et al. found that drop-out rates were approximately 25% after injections 1 and 2 and thereafter none.^36^ Discontinuation rates were also associated mainly with poor efficacy in 13% and CISC issues in 11%. Efficacy was maintained for up to five injections according to analyses with significant improvements in OAB symptoms and QoL parameters in this large prospective cohort. Khan et al prospectively followed 81 patients treated with 200U of onabotulinumtoxin A for IDO in a non-randomised, open label study.^30^ Significant improvements in symptom scores were noticed using the UDI-6 and the IIQ after up to five repeated injections. The overall CISC rate after treatment was 43% with residual volumes of over 100 ml being considered significant. Another study assessed altering the dose of the toxin in order to maximise efficacy and/or limit the need for CISC and reported on repeated injection outcomes.^37^ Patients had received up to four injections and significant improvements in OAB symptoms and QoL were observed after each injection as compared with baseline. Urodynamic parameters also improved with no evidence of reduced compliance. Nine patients had their BTX-A dose altered, with better outcomes in five.

## Cost effectiveness

In the UK a cost effectiveness analysis for onabotulinumtoxin A was conducted.^38^ Although a cost per quality-adjusted life year (QALY) gained calculation was not possible because of the lack of data linking bladder symptoms of DO to utility data needed to calculate QALYs gained, costings of the procedure were calculated based on National Health Service (NHS) standard costs and NHS resources used by typical patients. The overall costs of one set of onabotulinumtoxin A injections, including clinic consultation, basic investigations such as urine dipstick and an urodynamic study, the injection procedure with consumables, clinic review in the outpatient clinic post-injection with a further urine dipstick and post-void residual, equated to £745.33 for IDO and £874.62 for NDO.

## Conclusion

BoNT/A is now recognised as an effective therapeutic option to treat refractory DO. Over the last 10 years the UK has contributed significantly to the literature on the effects of this toxin in the clinical setting. Basic science research has given some valuable insight into its potential mechanism of action. It is hoped that soon it will receive a licence for OAB/IDO so that patients in need throughout the UK will have access to the beneficial effects of the toxin. Future study will concentrate on optimising its delivery to the bladder, assessing other bladder conditions beyond OAB in vigorous clinical trial settings and further developing an understanding of its complex mode of action.

## References

[1] NICE. The management of urinary incontinence in women. NICE 2006; Clinical Guideline, http://www.nice.org.uk/cg40 (2006, accessed, October).

[2] Thuroff JW Abrams P Andersson KE EAU guidelines on urinary incontinence. Eur Urol 2011; 59: 387–400. 21130559 10.1016/j.eururo.2010.11.021

[3] Apostolidis A Dasgupta P Denys P Recommendations on the use of botulinum toxin in the treatment of lower urinary tract disorders and pelvic floor dysfunctions: A European consensus report. Eur Urol 2009; 55: 100–119. 18823697 10.1016/j.eururo.2008.09.009

[4] Mangera A Andersson KE Apostolidis A Contemporary management of lower urinary tract disease with botulinum toxin A: A systematic review of botox (onabotulinumtoxinA) and dysport (abobotulinumtoxinA). Eur Urol 2011; 60: 784–795. 21782318 10.1016/j.eururo.2011.07.001

[5] Dolly JO Aoki KR The structure and mode of action of different botulinum toxins. Eur J Neurol 2006; 13 (Suppl 4): 1–9. 17112344 10.1111/j.1468-1331.2006.01648.x

[6] Apostolidis A Dasgupta P Fowler CJ Proposed mechanism for the efficacy of injected botulinum toxin in the treatment of human detrusor overactivity. Eur Urol 2006; 49: 644–650. 16426734 10.1016/j.eururo.2005.12.010

[7] Apostolidis A Popat R Yiangou Y Decreased sensory receptors P2X3 and TRPV1 in suburothelial nerve fibers following intradetrusor injections of botulinum toxin for human detrusor overactivity. J Urol 2005; 174: 977–982; discussion 982–973. 16094018 10.1097/01.ju.0000169481.42259.54

[8] Apostolidis A Jacques TS Freeman A Histological changes in the urothelium and suburothelium of human overactive bladder following intradetrusor injections of botulinum neurotoxin type A for the treatment of neurogenic or idiopathic detrusor overactivity. Eur Urol 2008; 53: 1245–1253. 18343564 10.1016/j.eururo.2008.02.037

[9] Chuang YC Huang CC Kang HY Novel action of botulinum toxin on the stromal and epithelial components of the prostate gland. J Urol 2006; 175: 1158–1163. 16469644 10.1016/S0022-5347(05)00318-6

[10] Chuang YC Giannantoni A Chancellor MB The potential and promise of using botulinum toxin in the prostate gland. BJU Int 2006; 98: 28–32. 16831138 10.1111/j.1464-410X.2006.06184.x

[11] McCloskey KD Interstitial cells in the urinary bladder — localization and function. Neurourol Urodyn 2010; 29: 82–87. 20025023 10.1002/nau.20739

[12] Wiseman OJ Fowler CJ Landon DN The role of the human bladder lamina propria myofibroblast. BJU Int 2003; 91: 89–93. 12614258 10.1046/j.1464-410x.2003.03802.x

[13] Roosen A Datta SN Chowdhury RA Suburothelial myofibroblasts in the human overactive bladder and the effect of botulinum neurotoxin type A treatment. Eur Urol 2009; 55: 1440–1448. 19054608 10.1016/j.eururo.2008.11.009

[14] Datta SN Roosen A Pullen A Immunohistochemical expression of muscarinic receptors in the urothelium and suburothelium of neurogenic and idiopathic overactive human bladders, and changes with botulinum neurotoxin administration. J Urol 2010; 184: 2578–2585. 21030043 10.1016/j.juro.2010.07.034

[15] Schurch B Stohrer M Kramer G Botulinum-A toxin for treating detrusor hyperreflexia in spinal cord injured patients: A new alternative to anticholinergic drugs? Preliminary results. J Urol 2000; 164: 692–697. 10953127 10.1097/00005392-200009010-00018

[16] Mehnert U Boy S Schmid M A morphological evaluation of botulinum neurotoxin A injections into the detrusor muscle using magnetic resonance imaging. World J Urol 2009; 27: 397–403. 19145439 10.1007/s00345-008-0362-0

[17] Popat R Apostolidis A Kalsi V A comparison between the response of patients with idiopathic detrusor overactivity and neurogenic detrusor overactivity to the first intradetrusor injection of botulinum-A toxin. J Urol 2005; 174: 984–989. 16094019 10.1097/01.ju.0000169480.43557.31

[18] Harper M Popat RB Dasgupta R A minimally invasive technique for outpatient local anaesthetic administration of intradetrusor botulinum toxin in intractable detrusor overactivity. BJU Int 2003; 92: 325–326. 12887493 10.1046/j.1464-410x.2003.04312.x

[19] Manecksha RP Cullen IM Ahmad S Prospective randomised controlled trial comparing trigone-sparing versus trigone-including intradetrusor injection of abobotulinumtoxinA for refractory idiopathic detrusor overactivity. Eur Urol 2012; 61: 928–935. 22078337 10.1016/j.eururo.2011.10.043

[20] Kalsi V Apostolidis A Popat R Quality of life changes in patients with neurogenic versus idiopathic detrusor overactivity after intradetrusor injections of botulinum neurotoxin type A and correlations with lower urinary tract symptoms and urodynamic changes. Eur Urol 2006; 49: 528–535. 16426735 10.1016/j.eururo.2005.12.012

[21] Kalsi V Apostolidis A Gonzales G Early effect on the overactive bladder symptoms following botulinum neurotoxin type A injections for detrusor overactivity. Eur Urol 2008; 54: 181–187. 18191323 10.1016/j.eururo.2007.12.029

[22] Patki PS Hamid R Arumugam K Botulinum toxin-type A in the treatment of drug-resistant neurogenic detrusor overactivity secondary to traumatic spinal cord injury. BJU Int 2006; 98: 77–82. 16831148 10.1111/j.1464-410X.2006.06192.x

[23] Hori S Patki P Attar KH Patients’ perspective of botulinum toxin-A as a long-term treatment option for neurogenic detrusor overactivity secondary to spinal cord injury. BJU Int 2009; 104: 216–220. 19220255 10.1111/j.1464-410X.2009.08368.x

[24] Brubaker L Richter HE Visco A Refractory idiopathic urge urinary incontinence and botulinum A injection. J Urol 2008; 180: 217–222. 18499184 10.1016/j.juro.2008.03.028 PMC2597793

[25] Kalsi V Gonzales G Popat R Botulinum injections for the treatment of bladder symptoms of multiple sclerosis. Ann Neurol 2007; 62: 452–457. 17890635 10.1002/ana.21209

[26] Cruz F Herschorn S Aliotta P Efficacy and safety of onabotulinumtoxinA in patients with urinary incontinence due to neurogenic detrusor overactivity: A randomised, double-blind, placebo-controlled trial. Eur Urol 2011; 60: 742–750. 21798658 10.1016/j.eururo.2011.07.002

[27] Khan S Panicker J Roosen A Complete continence after botulinum neurotoxin type A injections for refractory idiopathic detrusor overactivity incontinence: Patient-reported outcome at 4 weeks. Eur Urol 2010; 57: 891–896. 19394133 10.1016/j.eururo.2009.04.020

[28] Jeffery S Fynes M Lee F Efficacy and complications of intradetrusor injection with botulinum toxin A in patients with refractory idiopathic detrusor overactivity. BJU Int 2007; 100: 1302–1306. 17979928 10.1111/j.1464-410X.2007.07186.x

[29] Sahai A Khan MS Dasgupta P Efficacy of botulinum toxin-A for treating idiopathic detrusor overactivity: Results from a single center, randomized, double-blind, placebo controlled trial. J Urol 2007; 177: 2231–2236. 17509328 10.1016/j.juro.2007.01.130

[30] Khan S Kessler TM Apostolidis A What a patient with refractory idiopathic detrusor overactivity should know about botulinum neurotoxin type a injection. J Urol 2009; 181: 1773–1778. 19233414 10.1016/j.juro.2008.11.110

[31] Tincello DG Kenyon S Abrams KR Botulinum toxin a versus placebo for refractory detrusor overactivity in women: a randomised blinded placebo-controlled trial of 240 women (the RELAX study). Eur Urol 2012; 62: 507–514. 22236796 10.1016/j.eururo.2011.12.056

[32] Sahai A Khan MS Le Gall N Urodynamic assessment of poor responders after botulinum toxin-A treatment for overactive bladder. Urology 2008; 71: 455–459. 18342186 10.1016/j.urology.2007.11.039

[33] Sahai A Sangster P Kalsi V Assessment of urodynamic and detrusor contractility variables in patients with overactive bladder syndrome treated with botulinum toxin-A: Is incomplete bladder emptying predictable? BJU Int 2009; 103: 630–634. 18990156 10.1111/j.1464-410X.2008.08076.x

[34] Kessler TM Khan S Panicker J Clean intermittent self-catheterization after botulinum neurotoxin type A injections: Short-term effect on quality of life. Obstet Gynecol 2009; 113: 1046–1051. 19384119 10.1097/AOG.0b013e3181a1f5ea

[35] Mohee A Khan A Harris N Long-term outcome of the use of intravesical botulinum toxin for the treatment of overactive bladder (OAB). BJU Int. Epub ahead of print 6 June 2012. DOI: 10.1111/j.1464-410X.2012.11282.x. 22672569

[36] Dowson C Watkins J Khan MS Repeated botulinum toxin type A injections for refractory overactive bladder: Medium-term outcomes, safety profile, and discontinuation rates. Eur Urol 2012; 61: 834–839. 22204745 10.1016/j.eururo.2011.12.011

[37] Sahai A Dowson C Khan MS Repeated injections of botulinum toxin-A for idiopathic detrusor overactivity. Urology 2010; 75: 552–558. 20035984 10.1016/j.urology.2009.05.097

[38] Kalsi V Popat RB Apostolidis A Cost-consequence analysis evaluating the use of botulinum neurotoxin-A in patients with detrusor overactivity based on clinical outcomes observed at a single UK centre. Eur Urol 2006; 49: 519–527. 16413656 10.1016/j.eururo.2005.11.006

